# Influence of elevated CO_2_ on development and food utilization of armyworm *Mythimna separata* fed on transgenic *Bt* maize infected by nitrogen-fixing bacteria

**DOI:** 10.7717/peerj.5138

**Published:** 2018-07-05

**Authors:** Zhuo Li, Megha N. Parajulee, Fajun Chen

**Affiliations:** 1 Department of Entomology, Nanjing Agricultural University, Nanjing, China; 2 AgriLife Research and Extension Center, Texas A&M University, Lubbock, TX, USA

**Keywords:** Elevated CO_2_, Growth and development, Transgenic *Bt* maize, Food utilization, Rhizobacteria, *Mythimna separata*

## Abstract

**Background:**

*Bt* crops will face a new ecological risk of reduced effectiveness against target-insect pests owing to the general decrease in exogenous-toxin content in *Bt* crops grown under elevated carbon dioxide (CO_2_). The method chosen to deal with this issue may affect the sustainability of transgenic crops as an effective pest management tool, especially under future atmospheric CO_2_ level raising.

**Methods:**

In this study, rhizobacterias, as being one potential biological regulator to enhance nitrogen utilization efficiency of crops, was selected and the effects of *Bt* maize (Line IE09S034 with *Cry1Ie* vs. its parental line of non-*Bt* maize Xianyu 335) infected by *Azospirillum brasilense* (AB) and *Azotobacter chroococcum* (AC) on the development and food utilization of the target *Mythimna separate* under ambient and double-ambient CO_2_ in open-top chambers from 2016 to 2017.

**Results:**

The results indicated that rhizobacteria infection significantly increased the larval life-span, pupal duration, relative consumption rate and approximate digestibility of *M. separata*, and significantly decreased the pupation rate, pupal weight, adult longevity, fecundity, relative growth rate, efficiency of conversion of digested food and efficiency of conversion of ingested food of *M. separata* fed on *Bt* maize, while here were opposite trends in development and food utilization of *M. separata* fed on non-*Bt* maize infected with AB and AC compared with the control buffer in 2016 and 2017 regardless of CO_2_ level.

**Discussion:**

Simultaneously, elevated CO_2_ and *Bt* maize both had negative influence on the development and food utilization of *M. separata*. Presumably, CO_2_ concentration arising in future significantly can increase their intake of food and harm to maize crop; however, *Bt* maize infected with rhizobacterias can reduce the field hazards from *M. separata* and the application of rhizobacteria infection can enhance the resistance of *Bt* maize against target lepidoptera pests especially under elevated CO_2_.

## Introduction

With increased fossil fuel combustion and drastic changes in land utilization, the concentration of atmospheric carbon dioxide (CO_2_) has increased by more than 40%, from 280 to 400 ppm, between the industrial revolution and now (Ciais et al., 2013). The recent forecast indicated that atmospheric CO_2_ concentration will increase to approximately 900 ppm by 2,100 (Intergovernmental Panel on Climate Change (IPCC) 2014). Increasing atmospheric CO_2_ concentration alone can be very significant in crop production because of its direct effect on plant physiology and biochemistry (Cornelissen, 2011), and indirect effect on tri-trophic interactions involving plants, herbivores, and predators or pathogens (Robinson, Ryan & Newman, 2012; Trębicki et al., 2017). Elevated atmospheric CO_2_ also affects the crop production via direct or indirect impact on the physiology and feeding behavior of phytophagous insects (Zvereva & Kozlov, 2006; Massad & Dyer, 2010; O’Neill et al., 2010). These changes may then lead to more severe and frequent outbreaks of pest insects in agricultural ecosystems (Percy et al., 2002).

Several studies have shown that the elevated CO_2_ increased lepidopteran insect feeding and damage severity in agricultural crops (Ainsworth et al., 2007; Lindroth et al., 2001), because of the increased proportion of C:N in host plant tissue and lower nutritional quality caused by elevated CO_2_ (Ainsworth et al., 2007). For example, larvae of *Helicoverpa armigera* fed on wheat grown in elevated CO_2_ showed the extended larval life span and increased consumption with reduced growth rate (Chen, Wu & Ge, 2004). Transgenic maize that expresses insecticidal *Cry* proteins derived from the soil bacterium *Bacillus thuringiensis* Berliner (Bt) has been used to control target lepidopteran insects (Carrière, Crowder & Tabashnik, 2010; Huang et al., 2014; Walters et al., 2010), e.g., European corn borer *Ostrinia nubilalis* (Hübner), Asian corn borer *O. furnacalis* (Guenée) (Lepidoptera: Crambidae) and corn armyworm *Mythimna separata* (Lepidoptera: Noctuidae) (Guo et al., 2016; Zhang et al., 2013; Jia et al., 2016). Transgenic *Bt* maize has widely been adopted worldwide (Cattaneo et al., 2006; Huang et al., 2005; Hutchison et al., 2010; Lu et al., 2012). It was anticipated that the primary effect of elevated CO_2_ on *Bt* toxin production would be due to differences in N concentration in plant tissues (Coviella, Stipanovic & Trumble, 2002). Biologically relevant changes in plant defensive chemistry of *Bt* maize are expected to have measurable effects on the target lepidopteran pests under climate change.

Additionally, many researchers found that the nitrogen metabolism of transgenic *Bt* crops could affect the expression of *Bt* toxin protein, and stimulating plant N uptake to increase in biomass N relative to C to increase the nitrate reductase activity and *Bt* toxin production of *Bt* crops. (Stitt & Krapp, 1999; Pang et al., 2005; Gao et al., 2009). Nitrogen plays the most important role for plant growth, and it is an important complement of enzymes catalyzing and controlling reactions in plants for normal physiological processes (Richardson et al., 2009). While most of nitrogen in the environment is found in a form of nitrogen gas (N_2_) which approximately amounts to 78% in the atmosphere, plant available nitrogen found in soil is generally derived from fertilizer augmentation. As plants cannot use N_2_ directly, soil-inhabiting microbes play a significant role in nitrogen uptake by plants as they change the N_2_ into ammonia (Yamprai, Mala & Sinma, 2014). *Azospirillum* sp. and *Azotobacter* sp. are the two major free-living soil microbes (Biari, Gholami & Rahmani, 2008), that are economically important nitrogen-fixing bacteria in maize crop production system (Yamprai, Mala & Sinma, 2014). Thus, optimization of soil-nitrogen management offers significant potential in the utilization of soil rhizobacterias to increase *Bt*-crop nitrogen utilization to affect the expression of *Bt* toxin under elevated CO_2_.

## Materials and Methods

### Setup of CO_2_ levels

A two-year study (2016–2017) was conducted in six open-top chambers (i.e., OTCs; Granted Patent: ZL201120042889.1; 2.5 m in height × 3.2 m in diameter) (Chen et al., 2011) at the Innovation Research Platforms for Climate Change, Biodiversity and Pest Management (CCBPM; http://www.ccbpm.org) field laboratory in Ningjin County, Shandong Province of China (37°38′ 30.7″ N, 116°51′ 11.0″ E). A total of two CO_2_ levels, ambient (375 μl/L, hereafter referred to as *a*CO_2_) and elevated (750 μl/L or double-ambient, hereafter referred to as *e*CO_2_) were applied continuously from 10 June to 7 October in both years. A total of three OTCs were used for each CO_2_ treatment, and the CO_2_ concentrations in each OTC were monitored continuously and adjusted using an infrared CO_2_ analyzer (Ventostat 8102; Telaire Company, Goleta, CA, USA). The OTCs of elevated CO_2_ treatments were inflated with canned CO_2_ gas with 95% purity and automatically controlled by the same type of infrared CO_2_ analyzer (Chen, Wu & Ge, 2004). Actual mean CO_2_ concentrations and temperature throughout the entire experiment for both 2016 and 2017 are provided in Table 1.

**Table 1:** Actual mean (±SE) CO_2_ concentration and temperature in the open-top chambers (OTC) from seedling emergence to harvest of transgenic *Bt* maize and its parental line of non-*Bt* maize in 2016 and 2017.

Climate factors	OTC	2016	2017	Two-way ANOVAs (*F*/*P* values)
CO_2_ (μl/L)	*e*CO_2_ OTC	744.4 ± 3.3^a, A^	748.8 ± 4.5^a, A^	*F*_CO2_ = 399.32, *P* = 0.000
				*F*_Year_ = 2.85, *P* = 0.13
	*a*CO_2_ OTC	372.6 ± 4.7^b, A^	374.5 ± 3.8^b, A^	*F*_Interaction_ = 0.45, *P* = 0.52
Temperature (°C)	*e*CO_2_ OTC	26.01 ± 0.5^a, A^	26.12 ± 0.3^a, A^	*F*_CO2_ = 0.006, *P* = 0.94
				*F*_Year_ = 3.38, *P* = 0.10
	*a*CO_2_ OTC	25.99 ± 0.4^a, A^	26.11 ± 0.4^a, A^	*F*_Interaction_ = 0.057, *P* = 0.82

**Notes:**

OTC: ambient-CO_2_ OTC (*a*CO_2_ OTC) and elevated-CO_2_ OTC (*e*CO_2_ OTC). Different lowercase letters indicate significantly different between the *e*CO_2_ OTC and *a*CO_2_ OTC in same year by the Duncan test at *P* < 0.05, respectively.

A Not significantly different between 2016 and 2017 at same CO_2_ level or temperature by the Duncan test at *P* > 0.05, respectively.

### Plant materials

The *Bt* maize cultivar (Line IE09S034, hereafter referred to as Bt) and its non-*Bt* parental line (cv. Xianyu 335, referred to as Xy) were both obtained from the Institute of Crop Sciences, Chinese Academy of Agricultural Sciences. Both *Bt* and non-*Bt* lines used in this study had the similar maturity (approximately 102 d: from 10 June to 20 September) and were well adapted to the growing conditions of northern China (Guo et al., 2016; Zhang et al., 2013; Jia et al., 2016; Ling, 2010). Both maize accessions were planted in plastic buckets (diameter × height = 30 × 45 cm) filled with 20 kg autoclaved soil and 10 g compound fertilizer (N:P:K = 18:15:12), then placed them into chambers on 10 June each year.

### Soil nitrogen-fixing bacteria and infection of maize seeds

Lyophilized *Azospirillum brasilense* (strain number ACCC 10103) and *Azotobacter chroococcum* (strain number ACCC 10006) were provided by Agriculture Culture Collection of China (ACCC) in plastic tubes (3 cm in diameter and 15 cm in height) with bacterial growth medium. Both species of rhizobacterias were grown in liquid medium at 28 °C under continuous shaking (200 rpm) until they reached an absorbance of 1.008 (*A. brasilense*) and 1.005 (*A. chroococcum*) at a wavelength of 600 nm. Before inoculation, the culture was centrifuged, and the supernatant was discarded, and the pellet of cells was re-suspended in the liquid medium to a density of 10^8^ copies per milliliter. The seeds of both *Bt* and non-*Bt* maize were infected with *A. brasilense* and *A. chroococcum* cultures each, and the inoculation doses were all adjusted to a final volume of 10 ml for each seed. After inoculation, all the treated seeds were maintained under sterile laminar air flow for 2 h at 28 °C (Cassán et al., 2009). Bacteria inoculation treatments consisted of three types of rhizobacteria infection, including (1) seeds infected with *A. brasilense* (referred to as AB); (2) seeds infected with *A. chroococcum* (referred to as AC); and (3) non-infected seeds (control) treated with a final volume of buffer solution (referred to as CK). The entire experiment, thus, consisted of 12 treatments, including two CO_2_ levels (*a*CO_2_ and *e*CO_2_), two maize cultivars (Bt and Xy), and three rhizobacteria infections (AB, AC, and CK), replicate six time. Each pot serves as one replication. Specifically, six buckets for each maize cultivar (Bt and Xy) and three rhizobacteria inoculations (6 buckets per transgenic treatment × 2 transgenic treatments × 3 inoculation treatments = 36 buckets) were placed randomly in each CO_2_ chamber (ambient and double-ambient CO_2_), and three maize seeds were sown in each bucket at 2 cm soil depth. No pesticides were applied during the entire experimental period and the manual weeding keep the maize buckets weed-free during the experiment. The rhizosphere soil was sampled from each bucket one-day before planting, 14 days after planting, and at harvest and measured the relative density of *A. brasilense* and *A. chroococcum* using RT-PCR (Tables 2 and 3) (Jiang et al., 2017).

**Table 2:** Sequence specific primers of rhizobacterias, *Azospirillum brasilense* (AB) and *Azotobacter chroococcum* (AC) for qRT-PCR.

Primer	Sequence (5′-3′)	GenBank accession	Description
AB-4	Forward: CAAGGGCACCATCCCGAC	X51500.1	*A. brasilense* NifH gene
	Reverse: CTGCTGCTCCTCCGACT		
AC-2	Forward: GTGACCCGAAAGCTGACTCC	EU693338.1	*A. chroococcum* nifH gene
	Reverse: CCACCTTCAGCACGTCTTCC		

**Table 3:** The rhizosphere soil densities of rhizobacterias inoculated in the potted soil of transgenic *Bt* maize and its parental line of non-*Bt* maize grown under ambient and elevated CO_2_ in 2016 and 2017.

Measure matters	Rhizobacteria infections		2016 (AB; AC copies/g)	2017 (AB; AC copies/g)
Sampled soil before maize planting			5.53 ± 0.24 10^5^; 4.47 ± 0.12 10^5^	5.61 ± 0.11 10^5^; 4.33 ± 0.17 10^5^
Sampled soil at the maize seedling after 14 days	AB	*a*CO_2_-Bt	8.46 ± 0.24 10^11^; 4.48 ± 0.26 10^5^	8.40 ± 0.28 10^11^; 4.44 ± 0.11 10^5^
		*a*CO_2_-Xy	8.25 ± 0.26 10^11^; 4.21 ± 0.08 10^5^	8.69 ± 0.23 10^11^; 4.56 ± 0.22 10^5^
		*e*CO_2_-Bt	8.36 ± 0.19 10^11^; 4.43 ± 0.15 10^5^	8.59 ± 0.21 10^11^; 4.47 ± 0.17 10^5^
		*e*CO_2_-Xy	8.70 ± 0.27 10^11^; 4.58 ± 0.29 10^5^	8.24 ± 0.12 10^11^; 4.34 ± 0.27 10^5^
	AC	*a*CO_2_-Bt	5.54 ± 0.25 10^5^; 7.37 ± 0.29 10^11^	5.70 ± 0.28 10^5^; 7.40 ± 0.26 10^11^
		*a*CO_2_-Xy	5.73 ± 0.24 10^5^; 7.29 ± 0.17 10^11^	5.36 ± 0.22 10^5^; 7.66 ± 0.25 10^11^
		*e*CO_2_-Bt	5.62 ± 0.30 10^5^; 7.71 ± 0.15 10^11^	5.13 ± 0.04 10^5^; 7.32 ± 0.13 10^11^
		*e*CO_2_-Xy	5.46 ± 0.28 10^5^; 7.59 ± 0.17 10^11^	5.42 ± 0.13 10^5^; 7.57 ± 0.22 10^11^
	CK	*a*CO_2_-Bt	5.71 ± 0.20 10^5^; 4.52 ± 0.21 10^5^	5.92 ± 0.08 10^5^; 4.67 ± 0.17 10^5^
		*a*CO_2_-Xy	5.50 ± 0.29 10^5^; 4.24 ± 0.15 10^5^	5.33 ± 0.18 10^5^; 4.31 ± 0.13 10^5^
		*e*CO_2_-Bt	5.46 ± 0.08 10^5^; 4.26 ± 0.18 10^5^	5.62 ± 0.31 10^5^; 4.48 ± 0.21 10^5^
		*e*CO_2_-Xy	5.46 ± 0.18 10^5^; 4.76 ± 0.23 10^5^	5.47 ± 0.17 10^5^; 4.21 ± 0.09 10^5^
Sampled soil at the maize harvest	AB	*a*CO_2_-Bt	8.50 ± 0.19 10^11b^; 4.65 ± 0.21 10^5^	8.39 ± 0.26 10^11b^; 4.01 ± 0.26 10^5^
		*a*CO_2_-Xy	8.44 ± 0.15 10^11b^; 4.11 ± 0.23 10^5^	8.65 ± 0.19 10^11b^; 4.30 ± 0.18 10^5^
		*e*CO_2_-Bt	9.81 ± 0.23 10^11a^; 4.13 ± 0.17 10^5^	1.09 ± 0.04 10^12a^; 4.67 ± 0.20 10^5^
		*e*CO_2_-Xy	9.98 ± 0.25 10^11a^; 4.49 ± 0.22 10^5^	1.02 ± 0.03 10^12a^; 4.89 ± 0.23 10^5^
	AC	*a*CO_2_-Bt	5.57 ± 0.31 10^5^; 7.27 ± 0.26 10^11b^	5.40 ± 0.08 10^5^; 7.30 ± 0.14 10^11b^
		*a*CO_2_-Xy	5.99 ± 0.25 10^5^; 7.49 ± 0.19 10^11b^	4.97 ± 0.15 10^5^; 7.24 ± 0.19 10^11b^
		*e*CO_2_-Bt	4.89 ± 0.27 10^5^; 8.98 ± 0.15 10^11a^	5.94 ± 0.14 10^5^; 9.07 ± 0.12 10^11a^
		*e*CO_2_-Xy	5.33 ± 0.10 10^5^; 8.96 ± 0.21 10^11a^	5.77 ± 0.12 10^5^; 9.03 ± 0.18 10^11a^
	CK	*a*CO_2_-Bt	5.15 ± 0.35 10^5^; 4.65 ± 0.23 10^5^	5.39 ± 0.08 10^5^; 4.56 ± 0.22 10^5^
		*a*CO_2_-Xy	5.49 ± 0.19 10^5^; 4.37 ± 0.33 10^5^	4.97 ± 0.16 10^5^; 4.66 ± 0.15 10^5^
		*e*CO_2_-Bt	5.59 ± 0.14 10^5^; 4.76 ± 0.11 10^5^	4.88 ± 0.25 10^5^; 4.64 ± 0.17 10^5^
		*e*CO_2_-Xy	5.12 ± 0.14 10^5^; 4.69 ± 0.05 10^5^	5.13 ± 0.13 10^5^; 4.24 ± 0.10 10^5^

**Note:**

Rhizobacteria infections: *A. brasilense* (AB) and *A. chroococcum* (AC) vs. the control buffer solution (CK). CO_2_ levels: ambient CO_2_ (*a*CO_2_) and elevated CO_2_ (*e*CO_2_). Transgenic treatment: *Bt* maize (Bt) and non-*Bt* maize (Xy). Different lowercase letters indicate significantly different between ambient CO_2_ and elevated CO_2_ for same maize cultivar in same year by the Duncan test at *P* < 0.05, respectively.

### Insect source and rearing

The colony of armyworm *M. separata* was originated from a population collected in maize fields in Kangbao County, Hebei province of China (41.87°N, 114.6°E) in the summer of 2014, and fed on artificial diet and maintained for more than 10 generations in climate-controlled growth chambers (GDN-400D-4; Ningbo Southeast Instrument Co., Ltd., Ningbo, China) at 26 ± 1 °C, 65 ± 5% RH, and 14: 10 h L/D photoperiod. The same rearing conditions were maintained for the following experiments. Newly-hatched larvae were randomly selected from the above colony of *M. separata* and fed on artificial diet (Bi, 1981) until the second instar larvae, and then the third instar larvae were individually fed on excised leaves of the experimental plants growing in CO_2_ chambers. Feeding trials were conducted in plastic dish (6 cm in diameter and 1.6 cm in height) and the experimental leaves were randomly selected from six buckets for each of the 12 experimental treatment combinations (2 transgenic treatments × 2 CO_2_ treatments × 3 bacteria inoculations) during the tasseling stage until pupation. Sample size for the *M. separata* larval feeding trial consisted of 20 larvae (sample unit size) with five replicates for each of the 12 treatment combinations (i.e., 1,200 larvae evaluated for the entire study). Because of the cannibalism among the late instar larvae of *M. separata* (Jiang et al., 2016; Ali et al., 2016; Liu et al., 2017), the sampled larvae were reared separately in the Petri dish until pupation.

### Development and reproduction of *M. separata*

Larval development was evaluated from third instar to pupation by way of observing each individual petri dish every 8 h and recording the timing of larval ecdysis, pupation, and emergence of *M. separata* moths. After eclosion, the newly emerged moths were paired (female: male = 1:1) for mating in a metal frame screen cage (length × width × height = 35 × 35 × 40 cm), and the paired moths were fed with a 10% honey solution provided on a large cotton wick in a single plastic cup (diameter × height = 8 × 20 cm) covered with cotton net yarn butter paper for oviposition. The cotton net yarn and butter paper were replaced every day. Moth survivorship and oviposition were recorded daily until both moths from each pair died.

### Food utilization of the larvae of *M. separata*

Each third instar test larvae of *M. separate* was weighed at the initiation of the feeding trial by using an electronic balance (AL104; METTLER-TOLEDO, Greifensee, Switzerland). Total accumulated feces from third instar until the larva entered pupal stage (sixth instar), sixth instar larval weight, and the remaining leaves were also weighed. The food utilization indices of *M. separata* included the relative growth rate (RGR), relative consumption rate (RCR), approximate digestibility (AD), efficiency of conversion of ingested food (ECI) and efficiency of conversion of digested food (ECD) (Chen, Ge & Parajulee, 2005a; Chen et al., 2005b). Formulas for calculation of the measured indices were adapted from Chen et al. (2005b).

### Data analysis

All data were analyzed using the statistical software SPSS 19.0 (2015; SPSS Institute, Chicago, IL, USA). Four-way analysis of variance was used to analyze the effects of CO_2_ levels (elevated vs. ambient), transgenic treatment (*Bt* maize vs. non-*Bt* maize), rhizobacteria infection (AB and AC vs. CK), sampling years (2016 vs. 2017), and the interactions on the measured indices of growth, development, and reproduction, including larval life-span, pupation rate, pupal weight, pupal duration, adult longevity and fecundity of *M. separata*. The measured food utilization indices were analyzed by using an analysis of covariance with initial weight of *M. separata* (i.e., third instar larva) as a covariate for RCR and RGR, while food consumption was a covariate for ECI and AD to correct the effect of variation in the growth and food assimilation of *M. separata* (Raubenheimer & Simpson, 1992); food assimilated was also used as a covariate to analyze the ECD parameter (Hägele & Rowell-Rahier, 1999). The assumption of a parallel slope between covariate and dependent variable was satisfied for each analysis. Treatment means were separated by using the Duncan-test to examine significant difference at *P* < 0.05.

## Results

### Effects of CO_2_ level, transgenic treatment, and rhizobacteria infection on the rhizosphere soil densities of *A. brasilense* and *A. chroococcum* in different sampling period

Significant effects of rhizobacteria infection (*P* < 0.001) were observed on the measured rhizosphere soil densities of *A. brasilense* (AB) and *A. chroococcum* (AC) 14 days after maize planting. Compared with ambient CO_2_, elevated CO_2_ significantly increased the rhizosphere soil densities of both *A. brasilense* and *A. chroococcum*; compared with the control buffer solution (CK), rhizobacteria infection significantly increased the rhizosphere soil densities of *A. brasilense* and *A. chroococcum* (*P* < 0.001; Table 3). CO_2_ level and rhizobacteria infection both significantly affected the densities of *A. brasilense* and *A. chroococcum* in rhizosphere soil at maize harvest (Table 4).

**Table 4:** Four-way ANOVA on the rhizosphere soil densities of rhizobacterias inoculated in the potted soil of *Bt* maize and its parental line of non-*Bt* maize grown under ambient and elevated CO_2_ in 2016 and 2017.

Impact factors	Sampled soil at the maize seedling after 14 days		Sampled soil at the maize harvest	
	AB	AC	AB	AC
Y^a^	0.00/0.99	0.002/0.96	0.97/0.33	1.13/0.29
Cv.^b^	0.32/0.574	0.36/0.55	0.070/0.79	0.080/0.78
CO_2_^c^	0.19/0.89	0.80/0.38	26.01/<0.001^***^	331.16/<0.001^***^
Rhizobacteria^d^	2555.00/<0.001^***^	1380.37/<0.001^***^	1311.83/<0.001^***^	2080.71/<0.001^***^
Y × Cv.	0.62/0.44	1.96/0.17	0.18/0.673	0.53/0.47
Y × CO_2_	1.21/0.28	2.50/0.12	0.74/0.40	0.032/0.86
Y × Rhizobacteria	0.00/1.00	0.02/0.99	0.97/0.39	1.13/0.33
Cv. × CO_2_	0.35/0.55	0.010/0.92	0.32/0.57	0.36/0.55
Cv. × Rhizobacteria	0.32/0.73	0.36/0.70	0.070/0.93	0.80/0.93
CO_2_ × Rhizobacteria	0.019/0.98	0.80/0.45	26.01/<0.001^***^	331.16/<0.001^***^
Y × Cv. × CO_2_	5.99/0.018^*^	0.06/0.94	0.82/0.37	0.63/0.43
Y × Cv. × Rhizobacteria	0.62/0.54	1.96/0.15	0.18/0.84	0.53/0.59
Y × CO_2_ × Rhizobacteria	1.21/0.31	2.50/0.093	0.74/0.49	0.032/0.97
Cv. × CO_2_ × Rhizobacteria	0.35/0.70	0.10/0.99	0.32/0.73	0.36/0.70
Y × Cv. × CO_2_ × Rhizobacteria	5.99/0.05	0.006/0.99	0.82/0.45	0.63/0.54

**Notes:**

* *P* < 0.05;

** *P* < 0.01;

*** *P* < 0.001.

a Year (2016 vs. 2017).

b Transgenic treatment (*Bt* maize vs. non-*Bt* maize).

c CO_2_ levels (elevated CO_2_ vs. ambient CO_2_).

d Rhizobacteria infection (*A. brasilense* and *A. chroococcum* vs. the control buffer), the same as in Tables 5 and 7.

### Effects of CO_2_ level, transgenic treatment, and rhizobacteria infection on the development and reproduction of *M. separata*

Carbon dioxide level and transgenic treatment both significantly affected the larval life-span, pupation rate, pupal weight and duration, adult longevity, and fecundity in *M. separata* fed on both *Bt* and non-*Bt* maize infected with *A. brasilense* and *A. chroococcum* (*P* < 0.001). However, the rhizobacteria infection significantly affected the larval life-span, pupal duration (*P* < 0.05) and fecundity (*P* < 0.001) of *M. separata* fed on both transgenic treatments and at both CO_2_ levels (Table 5).

**Table 5:** Four-way ANOVA on the development and reproduction of *Mythimna separata* fed on *Bt* and non-*Bt* maize infected with *A. brasilense* and *A. chroococcum* under ambient and elevated CO_2_ in 2016 and 2017.

Impact factors	Larval life-span (day (*n* = 828))	Pupation rate (% (*n* = 828))	Pupal weight (g (*n* = 663))	Pupal duration (day (*n* = 663))	Adult longevity (day (*n* = 576))	Fecundity (eggs per female (*n* = 198))
Y^a^	1.62/0.32	0.94/0.71	1.21/0.62	0.11/0.90	1.65/0.33	2.99/0.10
Cv.^b^	1662.03/<0.001^***^	329.16/<0.001^***^	275.04/<0.001^***^	229.78/<0.001^***^	450.27/<0.001^***^	2032.99/<0.001^***^
CO_2_^c^	62.13/<0.001^***^	55.53/<0.001^***^	12.44/0.001^**^	19.02/<0.001^***^	41.62/<0.001^***^	278.70/<0.001^***^
Rhizobacteria^d^	3.64/0.034^*^	2.53/0.077	1.20/0.63	7.04/0.011^*^	0.35/0.70	27.54/<0.001^***^
Y × Cv.	9.22/0.004^**^	2.22/0.14	2.22/0.14	1.98/0.32	3.10/0.054	0.102/0.75
Y × CO_2_	5.33/0.025^*^	2.16/0.20	0.067/0.80	0.17/0.85	13.53/<0.001^***^	2.74/0.10
Y × Rhizobacteria	3.63/0.034^*^	1.69/0.058	0.63/0.57	0.48/0.71	0.59/0.64	1.87/0.053
Cv. × CO_2_	19.11/<0.001^***^	6.62/<0.001^***^	2.68/0.008^**^	14.11/<0.001^***^	8.26/0.006^**^	5.86/0.049^*^
Cv. × Rhizobacteria	224.53/<0.001^***^	73.67/<0.001^***^	30.41/<0.001^***^	42.06/<0.001^**^	26.38/<0.001^***^	195.08/<0.001^***^
CO_2_ × Rhizobacteria	12.81/<0.001^***^	12.22/0.004^**^	11.63/0.005^**^	14.98/<0.001^***^	4.24/0.02^*^	22.02/<0.001^***^
Y × Cv. × CO_2_	0.01/0.92	1.07/0.55	0.10/0.75	1.16/0.52	0.006/0.94	4.25/0.085
Y × Cv. × Rhizobacteria	1.55/0.33	1.53/0.17	0.064/0.94	2.02/0.23	0.46/0.68	8.87/0.071
Y × CO_2_ × Rhizobacteria	0.063/0.69	0.51/0.76	0.48/0.62	1.72/0.45	1.78/0.18	14.61/0.045^*^
Cv. × CO_2_ × Rhizobacteria	8.88/0.006^**^	12.61/0.003^**^	7.41/0.011^*^	5.66/0.005^**^	13.55/0.002^**^	24.04/0.000^***^
Y × Cv. × CO_2_ × Rhizobacteria	0.19/0.83	0.33/0.71	0.14/0.87	0.32/0.72	1.73/0.30	0.36/0.70

**Notes:**

* *P* < 0.05;

** *P* < 0.01;

*** *P* < 0.001.

a Year (2016 vs. 2017).

b Transgenic treatment (*Bt* maize vs. non-*Bt* maize).

c CO_2_ levels (elevated CO_2_ vs. ambient CO_2_).

d Rhizobacteria infection (*A. brasilense* and *A. chroococcum* vs. the control buffer), the same as in Tables 4 and 7.

Compared with ambient CO_2_, elevated CO_2_ significantly prolonged the larval life-span (+6.21%), pupal duration (+5.56%), and significantly decreased the pupation rate (−18.08%), pupal weight (−8.12%), adult longevity (−6.06%), and fecundity (−22.58%) of *M. separata* (*P* < 0.05; Table 6). Also, compared with the CK, rhizobacteria infection with *A. brasilense* and *A. chroococcum* both significantly shortened the larval life-span (−5.20% and −5.70%), pupal duration (−3.68% and −3.81%) and fecundity (−10.20% and −9.53%) of *M. separata* (*P* < 0.05; Table 6). Moreover, *Bt* maize significantly prolonged the larval life-span (+13.67%) and pupal duration (+7.54%), shortened the adult longevity (−10.41%), and decreased the pupation rate (−75.55%), pupal weight (−13.54%) and fecundity (−75.46%) of *M. separata* compared to that for non-*Bt* maize (*P* < 0.05; Table 6).

**Table 6:** The development and reproduction of *M. separata* larvae fed on *Bt* maize and non-*Bt* maize during the heading stage, infected with rhizobacterias under ambient and elevated CO_2_ in 2016 and 2017.

Impact factors	Factor levels	Larval life-span (day)	Pupation rate (%)	Pupal weight (g)	Pupal duration (day)	Adult longevity (day)	Fecundity (eggs per female)
Cv.	Bt	24.19 ± 0.16^a^	40.98 ± 2.45^b^	0.192 ± 0.007^b^	10.98 ± 0.18^a^	6.63 ± 0.14^b^	171.50 ± 13.27^b^
	Xy	21.28 ± 0.17^b^	71.94 ± 2.38^a^	0.218 ± 0.006^a^	10.21 ± 0.19^b^	7.32 ± 0.09^a^	300.92 ± 28.64^a^
CO_2_	Elevated	23.42 ± 0.20^a^	51.78 ± 2.69^b^	0.197 ± 0.007^b^	10.78 ± 0.18^a^	6.77 ± 0.11^b^	212.25 ± 19.13^b^
	Ambient	22.05 ± 0.17^b^	61.14 ± 2.68^a^	0.213 ± 0.005^a^	10.41 ± 0.19^b^	7.18 ± 0.13^a^	260.17 ± 21.87^a^
Rhizobacteria infection	AB	22.53 ± 0.16^b^	56.06 ± 2.24	0.205 ± 0.004	10.54 ± 0.09^b^	6.97 ± 0.24	228.00 ± 15.27^b^
	AC	22.42 ± 0.15^b^	55.53 ± 2.01	0.204 ± 0.004	10.53 ± 0.08^b^	6.96 ± 0.24	229.38 ± 17.46^b^
	CK	23.25 ± 0.16^a^	57.79 ± 2.58	0.206 ± 0.005	10.72 ± 0.13^a^	6.99 ± 0.18	251.25 ± 23.21^a^

**Note:**

Data in table are average ± SE. Different lowercase letters indicate significant difference between treatments by the Duncan’s test at *P* < 0.05; the same as in Table 8.

### Impacts of CO_2_ level, transgenic treatment, and rhizobacteria infection on the food utilization of *M. separata*

There were significant effects of CO_2_ level, transgenic treatment, and rhizobacteria infection (*P* < 0.01 or *P* < 0.001) on food utilization of *M. separata* fed on both *Bt* and non-*Bt* maize infected with *A. brasilense* and *A. chroococcum* at both CO_2_ levels in both years of the study (Table 7).

**Table 7:** Four-way ANCOVA on the food utilization indices of *Mythimna separata* fed on *Bt* maize and non-*Bt* maize infected with *A. brasilense* and *A. chroococcum* under ambient and elevated CO_2_ in 2016 and 2017.

Impact factors	The 3rd to 6th instar larvae (*n* = 828)				
	RGR (mg g^−1^day^−1^)	RCR (mg g^−1^day^−1^)	AD (%)	ECD (%)	ECI (%)
Covariate^e^	1.81/0.11	3.83/0.067	0.781/0.23	0.580/0.41	0.87/0.32
Y^a^	1.19/0.31	3.96/0.12	4.56/0.061	4.82/0.059	5.80/0.053
Cv.^b^	1545.53/<0.001^***^	302.67/0.<0.001^***^	185.62/0.<0.001^***^	716.17/0.<0.001^***^	1038.95/0.<0.001^***^
CO_2_^c^	67.09/<0.001^***^	27.98/<0.001^***^	9.69/0.003^**^	35.90/<0.001^***^	57.98/<0.001^***^
Rhizobacteria^d^	12.26/<0.001^***^	26.84/<0.001^***^	35.28/<0.001^***^	13.64/<0.001^***^	7.22/0.002^**^
Y × Cv.	4.27/0.049^*^	5.69/0.021^*^	1.86/0.18	19.21/<0.001^***^	15.64/<0.001^***^
Y × CO_2_	6.90/0.012^*^	4.82/0.033^*^	6.73/0.013^*^	6.22/0.016^*^	3.41/0.071
Y × Rhizobacteria	5.04/0.010^*^	0.17/0.84	0.27/0.77	0.436/0.65	0.25/0.78
Cv. × CO_2_	30.44/<0.001^***^	7.39/0.009^**^	1.40/0.043^*^	1.80/0.017^*^	1.61/0.011^*^
Cv. × Rhizobacteria	213.46/<0.001^***^	48.31/<0.001^***^	73.42/<0.001^***^	132.82/<0.001^***^	144.91/<0.001^***^
CO_2_ × Rhizobacteria	13.25/<0.001^***^	6.70/0.003^**^	9.78/<0.001^***^	13.01/<0.001^***^	12.63/<0.001^***^
Y × Cv. × CO_2_	0.220/0.64	1.83/0.18	4.16/0.047^*^	0.743/0.39	0.56/0.46
Y × Cv. × Rhizobacteria	3.27/0.047^*^	0.55/0.58	2.23/0.12	4.01/0.025^*^	1.41/0.25
Y × CO_2_ × Rhizobacteria	0.72/0.49	1.88/0.16	1.33/0.28	2.66/0.080	2.47/0.095
Cv. × CO_2_ × Rhizobacteria	0.62/0.043^*^	13.24/<0.001^***^	9.84/<0.001^***^	9.59/<0.001^***^	9.92/<0.001^***^
Y × Cv. × CO_2_ × Rhizobacteria	0.48/0.62	0.087/0.92	0.98/0.38	0.59/0.56	0.06/0.94

**Notes:**

* *P* < 0.05;

** *P* < 0.01;

*** *P* < 0.001.

a Year (2016 vs. 2017).

b Transgenic treatment (*Bt* maize vs. non-*Bt* maize).

c CO_2_ levels (elevated CO_2_ vs. ambient CO_2_).

d Rhizobacteria infection (*A. brasilense* and *A. chroococcum* vs. the control buffer), the same as in Tables 4 and 5.

e Initial weight as a covariate for RGR and RCR, and food consumption as a covariate for AD and ECI, and food assimilated as a covariate for ECD.

Compared with ambient CO_2_, elevated CO_2_ significantly reduced the RGR (−9.95%), ECD (−16.05%), and ECI (−17.95%), and significantly enhanced the RCR (+10.44%) and AD (+5.59%) of *M. separata* (*P* < 0.05; Table 8). Compared with the CK, rhizobacteria infection with *A. brasilense* and *A. chroococcum* both significantly decreased the ECD (−9.28% and −7.48%) and ECI (−9.22% and −7.91%), and significantly increased the RGR (+4.75% and +5.56%), RCR (+6.78% and +7.53%) and AD (+5.28% and +4.93%) in *M. separata* (*P* < 0.01; Table 8). Moreover, significant decreases in RGR (−13.85%), ECD (−41.25%) and ECI (−31.97%), and significant increases in RCR (+16.60%) and AD (+7.88%) were found when *M. separata* fed on *Bt* maize compared to that on non-*Bt* maize (*P* < 0.05; Table 8).

**Table 8:** The food utilization of *M. separata* larvae fed on *Bt* maize and non-*Bt* maize during the heading stage, infected with rhizobacterias under ambient and elevated CO_2_ in 2016 and 2017.

Impact factors	Factor levels	RGR (mg g^−1^day^−1^)	RCR (mg g^−1^day^−1^)	AD (%)	ECD (%)	ECI (%)
Cv.	Bt	82.79 ± 6.43^b^	1636.31 ± 13.23^a^	56.13 ± 0.72^a^	9.26 ± 0.98^b^	5.13 ± 0.57^b^
	Xy	94.26 ± 7.16^a^	1403.36 ± 14.80^b^	52.03 ± 0.69^b^	13.08 ± 1.23^a^	6.77 ± 0.66^a^
CO_2_	Elevated	84.33 ± 8.67^b^	1595.24 ± 16.14^a^	55.55 ± 0.87^a^	10.34 ± 1.52^b^	5.46 ± 0.76^b^
	Ambient	92.72 ± 6.59^a^	1444.43 ± 15.01^b^	52.61 ± 0.81^b^	12.00 ± 1.21^a^	6.44 ± 0.63^a^
Rhizobacteria infection	AB	89.64 ± 5.52^a^	1549.07 ± 9.32^a^	55.06 ± 0.85^a^	10.78 ± 1.35^b^	5.75 ± 0.67^b^
	AC	90.34 ± 5.52^a^	1559.90 ± 9.32^a^	54.88 ± 0.85^a^	10.96 ± 1.35^b^	5.82 ± 0.67^b^
	CK	85.58 ± 6.14^b^	1450.73 ± 9.54^b^	52.30 ± 0.62^b^	11.78 ± 1.13^a^	6.28 ± 0.66^a^

### Interactive influence of CO_2_ level, transgenic treatment, and rhizobacteria infection on growth, development and reproduction of *M. separata*

In addition to the significant main effects of CO_2_ level, transgenic treatment, and rhizobacteria infection, there were significant two-way and three-way interaction of these three main effects on larval life-span, pupation rate, pupal weight and duration, adult longevity, and fecundity of *M. separata* fed on *Bt* and non-*Bt* maize infected with *A. brasilense* and *A. chroococcum* under both CO_2_ levels in both years of the study (*P* < 0.05, *P* < 0.01 or *P* < 0.001; Table 5).

#### Transgenic treatment × CO_2_

**Figure 1:**
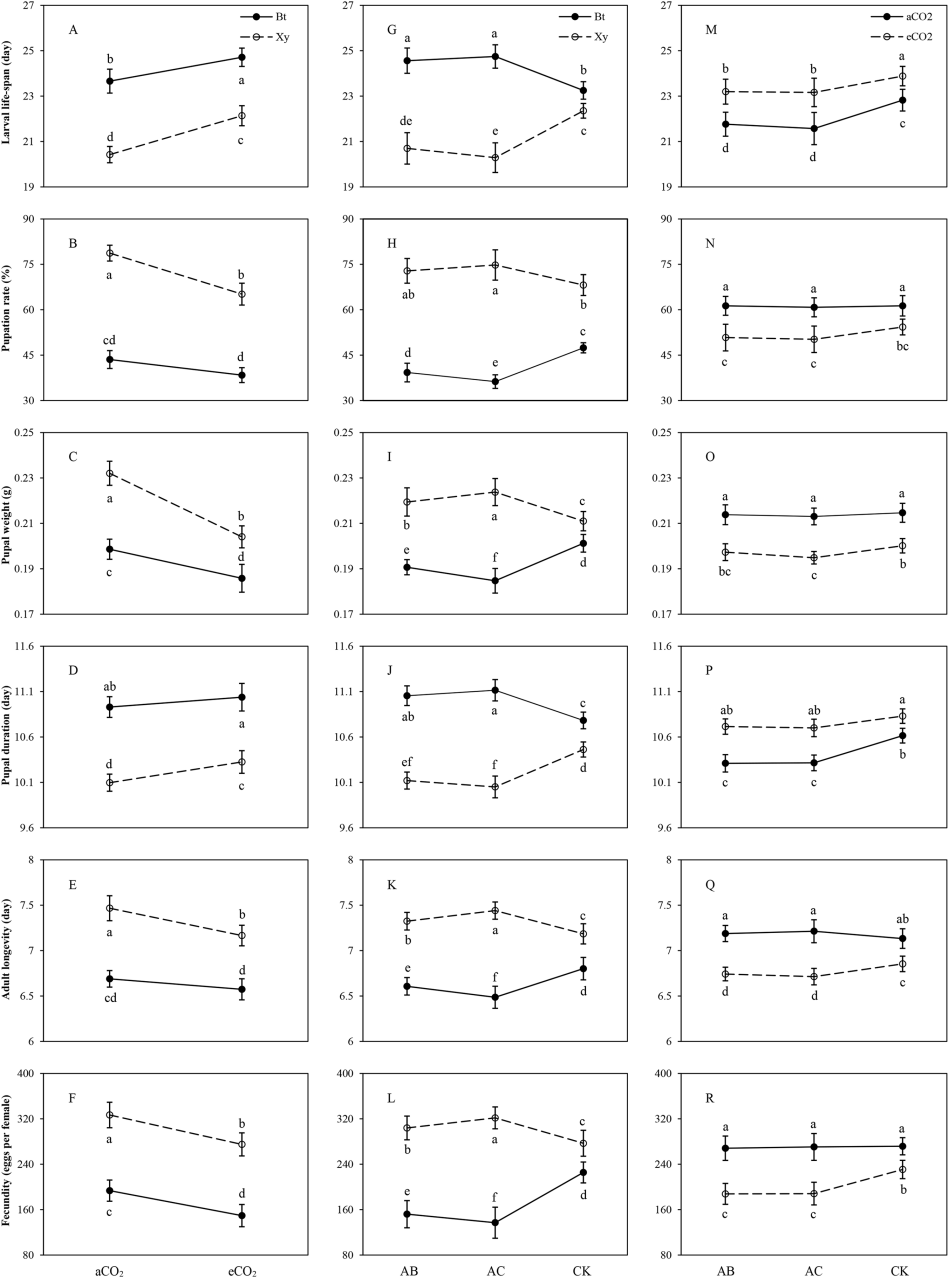
Effects of bi-interactions between transgenic treatment and CO_2_, between transgenic treatment and rhizobacteria and between CO_2_ and rhizobacteria on development and reproduction of *Mythimna separata*. Larval life-span–(A), (G), (M); Pupation rate–(B), (H), (N); Pupal weight–(C), (I), (O); Pupal duration–(D), (J), (P); Adult longevity–(E), (K), (Q); Fecundity–(F), (L), (R); Each value represents the average (±SE). Different lowercase letters indicate significant differences treatments by the Duncan test at *P* < 0.05.

Similar trends were found in the measured growth, development and reproduction indexes of *M. separata* fed on both *Bt* and non-*Bt* maize cultivars grown under elevated CO_2_ in contrast to ambient CO_2_, infected with *A. brasilense* (AB) and *A. chroococcum* (AC) as well as the CK in 2016 and 2017 (Figs. 1A–1F). Compared with ambient CO_2_, elevated CO_2_ significantly prolonged the larval life-span (*Bt* maize: +6.44%; non-*Bt* maize: +8.39%) and pupal duration (non-*Bt* maize: +7.27%) and shortened the adult longevity (non-*Bt* maize: −6.19%), and significantly decreased the pupation rate (non-*Bt* maize: −20.81%), pupal weight (*Bt* maize: −7.03%; non-*Bt* maize: −13.73%) and fecundity (*Bt* maize: −29.43%; non-*Bt* maize: −18.85%) when *M. separata* fed on *Bt* maize and non-*Bt* maize (*P* < 0.05; Figs. 1A–1F).

#### Transgenic treatment × Rhizobacteria

An inverse trend was found in the measured growth, development and reproduction indexes of *M. separata* fed on *Bt* maize and non-*Bt* maize, which were infected with *A. brasilense* (AB) and *A. chroococcum* (AC) under ambient and elevated CO_2_ in 2016 and 2017 (Figs. 1G–1L). Compared with the CK, rhizobacteria infection significantly prolonged the larval life-span (AB: +7.63%; AC: +8.45%), pupal duration (AB: +4.53%; AC: +5.08%) and shortened the adult longevity (AB: −4.88%; AC: −6.94%), and decreased pupation rate (AB: −20.83%; AC: −30.81%), pupal weight (AB: −7.24%; AC: −10.65%) and fecundity (AB: −48.36%; AC: −64.60%) when *M. separata* larvae fed on *Bt* maize (*P* < 0.01; Figs. 1G–1L); and rhizobacteria infection significantly shortened the larval life-span (AB: −7.97%; AC: −10.15%), pupal duration (AB: −5.36%; AC: −6.08%) and prolonged the adult longevity (AB: +3.95%; AC: +5.62%), and increased pupation rate (AC: +9.73%), pupal weight (AB: +6.27%; AC: +8.16%) and fecundity (AB: +9.75%; AC: +16.16%) when *M. separata* larvae fed on non-*Bt* maize (*P* < 0.01; Figs. 1G–1L).

#### CO_2_ × Rhizobacteria

Similar trends were found in the larval life-span, pupation rate, pupal weight, and pupal duration, while inverse trends were observed in adult longevity and fecundity of *M. separata* under ambient and elevated CO_2_, which fed on *Bt* maize vs. non-*Bt* maize infected with *A. brasilense* (AB) and *A. chroococcum* (AC) as well as the CK in 2016 and 2017 (Figs. 1M–1R). Compared with the CK, rhizobacteria infection significantly shortened the larval life-span (AB: −6.92%; AC: −7.84%) and pupal duration (AB: −5.01%; AC: −5.01%) of *M. separata* under ambient CO_2_, and significantly shortened the larval life-span (AB: −4.98%; AC: −5.11%) and decreased the pupal weight (AC: −4.77%) under elevated CO_2_ (*P* < 0.05; Figs. 1M–1P); and rhizobacteria infection significantly decreased the adult longevity (AB: −4.63%; AC: −5.09%) and fecundity (AB: −22.90%; AC: −22.58%) of *M. separata* under elevated CO_2_ (*P* < 0.05; Figs. 1Q and 1R).

#### Transgenic treatment × CO_2_ × Rhizobacteria

**Figure 2:**
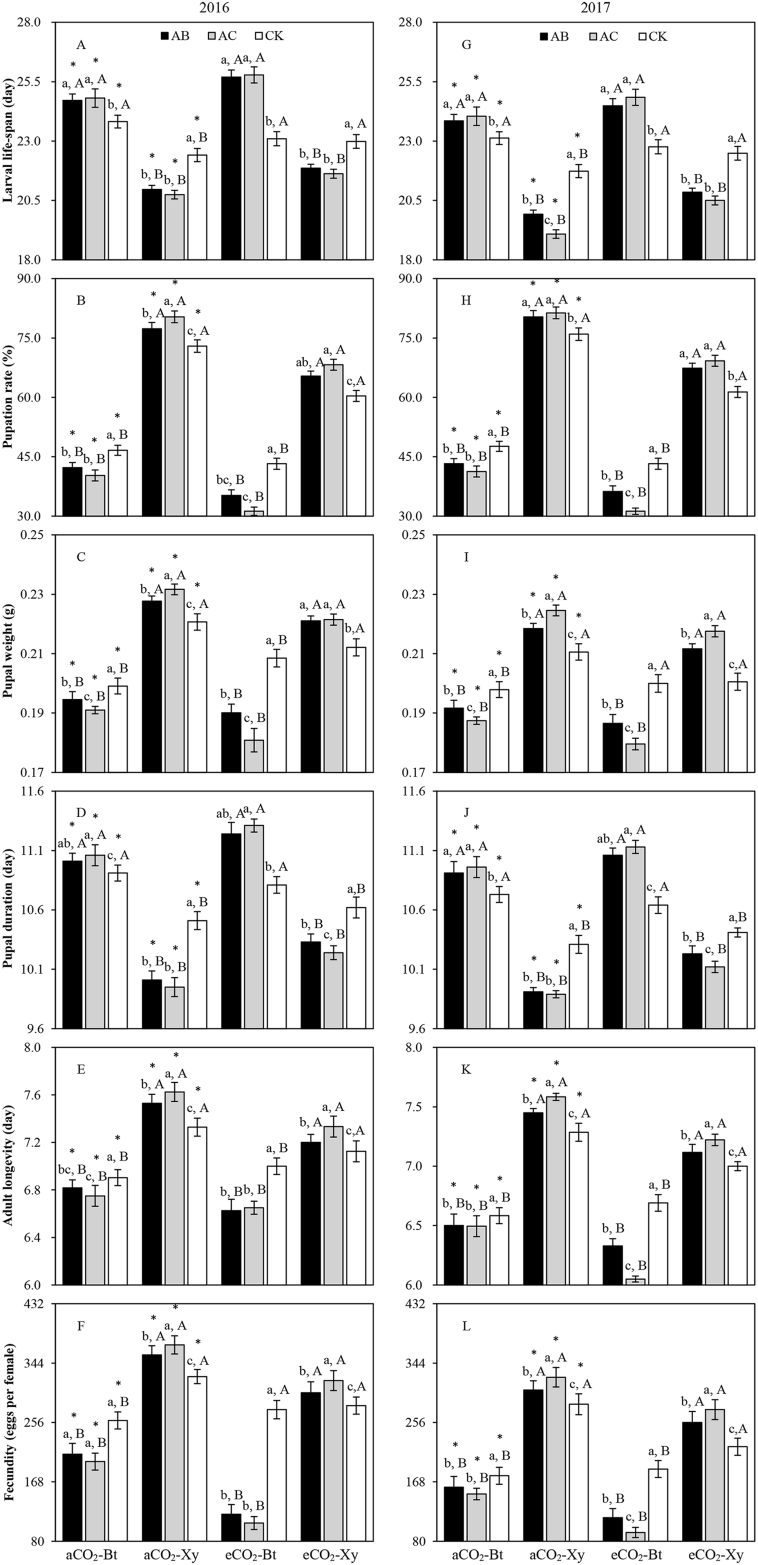
Impacts of the tri-interactions among CO_2_ level, transgenic treatment, and rhizobacteria infection on the growth, development and reproduction of *M. separata* in 2016 (A–F) and 2017 (G–L). Each value represents the average (+SE). Different lowercase and uppercase letters, and *indicated significant difference among three types of rhizobacteria infection for same type of maize under same CO_2_ level, between *Bt* maize and non-*Bt* maize for same type of rhizobacteria infection under same CO_2_ level, and between ambient and elevated CO_2_ for same type of maize and rhizobacteria infection by the Duncan test at *P* < 0.05 respectively.

There were opposite trends in the measured growth, development and reproduction indexes of *M. separata* fed on *Bt* maize and non-*Bt* maize infected with *A. brasilense* (AB) and *A. chroococcum* (AC) compared with the CK in 2016 and 2017 regardless of CO_2_ level (Fig. 2). In comparison with the CK, rhizobacteria infection with *A. brasilense* and *A. chroococcum* both significantly prolonged the larval life-span and pupal duration of *M. separata* fed on *Bt* maize, and significantly shortened the larval life-span and pupal duration of *M. separata* fed on non-*Bt* maize under the same CO_2_ level; and rhizobacteria infection with *A. brasilense* and *A. chroococcum* both significantly reduced the pupation rate, pupal weight, adult longevity and fecundity of *M. separata* fed on *Bt* maize, and significantly enhanced the pupation rate, pupal weight, adult longevity and fecundity of *M. separata* fed on non-*Bt* maize under the same CO_2_ level. Moreover, compared with ambient CO_2_, there were opposite trends in the larval life-span, pupal weight, pupal duration, adult longevity and fecundity of *M. separata* fed on *Bt* maize infected with *A. brasilense* and *A. chroococcum* compared with the CK under elevated CO_2_ in both years; compared with ambient CO_2_, elevated CO_2_ significantly decreased the pupation rate of *M. separata* fed on *Bt* maize, and decreased the pupation rate, pupal weight, adult longevity and fecundity of *M. separata* fed on non-*Bt* maize, and prolonged the larval life-span and pupal duration of *M. separata* fed on non-*Bt* maize infected with *A. brasilense* and *A. chroococcum* compared with the CK in both years.

### Interactive effects of CO_2_ level, transgenic treatment, and rhizobacteria infection on food utilization of *M. separata*

In addition to significant main effects of CO_2_ level, transgenic treatment, and rhizobacteria infection, two- and three-way interactions of these factors influenced the RGR, RCR, AD, ECD, and ECI of *M. separata* larvae fed on *Bt* maize and non-*Bt* maize infected with *A. brasilense* and *A. chroococcum* under ambient and elevated CO_2_ in both years (*P* < 0.05, *P* < 0.01 or *P* < 0.001; Table 7).

#### Transgenic treatment × CO_2_

**Figure 3:**
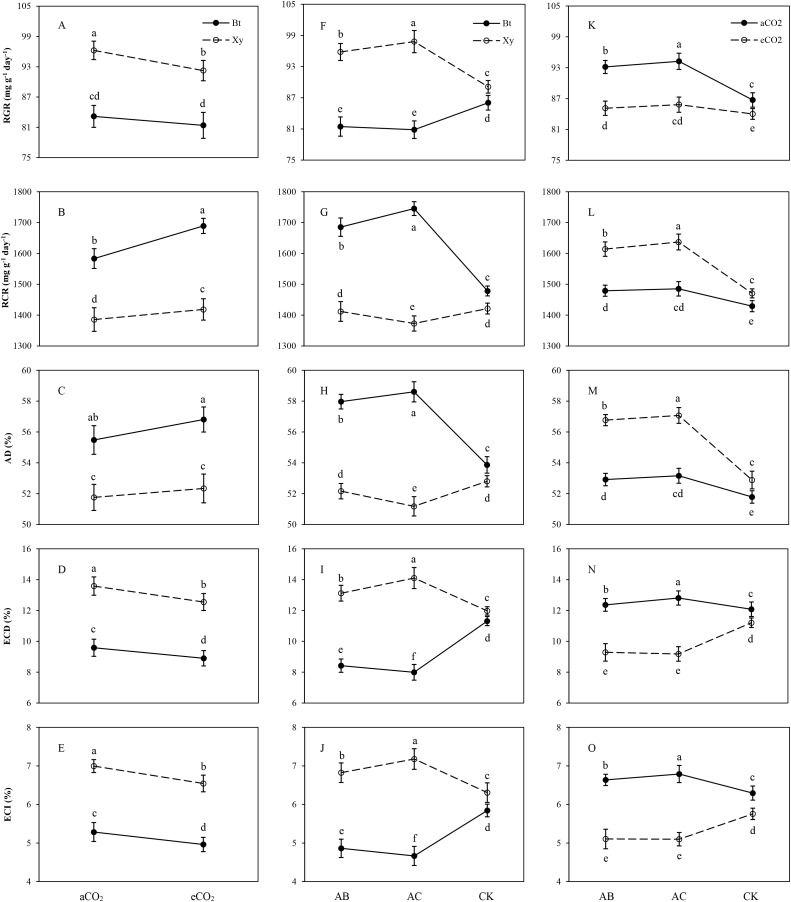
Effects of bi-interactions between transgenic treatment and CO_2_, between transgenic treatment and rhizobacteria and between CO_2_ and rhizobacteria on food utilization of *Mythimna separata* larvae. RGR–(A), (F), (K); RCR–(B), (G), (L); AD–(C), (H), (M); ECD–(D), (I), (N); ECI–(E), (J), (O); Each value represents the average (±SE). Different lowercase letters indicate significant differences treatments by the Duncan test at *P* < 0.05.

Similar trends were found in the measured food utilization indexes of *M. separata* fed on *Bt* maize (Bt) and non-*Bt* maize (Xy) grown under elevated CO_2_ in contrast to ambient CO_2_, infected with *A. brasilense* (AB) and *A. chroococcum* (AC) as well as the CK in 2016 and 2017 (Figs. 3A–3E). Compared with ambient CO_2_, elevated CO_2_ significantly decreased the RGR (non-*Bt* maize: −7.34%), ECD (*Bt* maize: −9.67%; non-*Bt* maize: −10.25%) and ECI (*Bt* maize: −8.53%; non-*Bt* maize: −8.89%), and significantly increased the RCR (*Bt* maize: +9.69%; non-*Bt* maize: +6.37%) when *M. separata* larvae fed on *Bt* maize and non-*Bt* maize (*P* < 0.05; Figs. 3A–3E).

#### Transgenic treatment × Rhizobacteria

Inverse trend was found in the measured food utilization indexes of *M. separata* fed on *Bt* maize and non-*Bt* maize, which were infected with *A. brasilense* (AB) and *A. chroococcum* (AC) under ambient and elevated CO_2_ in 2016 and 2017 (Figs. 3F–3J). Compared with the CK, rhizobacteria infection significantly enhanced the RGR (AB: +9.53%; AC: +11.78%), ECD (AB: +11.61%; AC: +19.79%) and ECI (AB: +10.08%; AC: +15.79%), and significantly decreased the RCR (AC: −6.52%) and AD (AC: −6.19%) when *M. separata* larvae fed on non-*Bt* maize (*P* < 0.001; Figs. 3F–3J); and rhizobacteria infection significantly decreased the RGR (AB: −9.62%; AC: −10.41%), ECD (AB: −34.32%; AC: −41.55%) and ECI (AB: −20.16%; AC: −25.28%), and significantly increased the RCR (AB: +14.99%; AC: +19.06%) and AD (AB: +9.60%; AC: +10.79%) when *M. separata* larvae fed on *Bt* maize (*P* < 0.001; Figs. 3F–3J).

#### CO_2_ × Rhizobacteria

Similar trends were observed in RGR, RCR, and AD, while inverse trends were shown in ECD and ECI of *M. separata* under ambient and elevated CO_2_, which fed on *Bt* maize and non-*Bt* maize infected with *A. brasilense* (AB) and *A. chroococcum* (AC) vs. CK (Figs. 3K–3O). Compared with the CK, rhizobacteria infection significantly decreased ECD (AB: −20.71%; AC: −22.07%) and ECI (AB: −12.77%; AC: −12.89%) of *M. separata* larvae under elevated CO_2_, and significantly increased ECD (AB: +5.35%; AC: +8.04%) and ECI (AB: +7.43%; AC: +9.85%) of *M. separata* larvae under ambient CO_2_ (*P* < 0.05; Fig. 3); and rhizobacteria infection significantly enhanced RGR (AB: +3.32% and +7.40%; AC: +5.14% and +8.67%), RCR (AB: +9.78% and +5.29%,; AC: +11.32% and +5.93%) and AD (AB: +7.34% and +4.18%; AC: +7.92% and +4.66%) under elevated and ambient CO_2_, respectively (*P* < 0.01; Figs. 3K–3O).

#### Transgenic treatment × CO_2_ × Rhizobacteria

**Figure 4:**
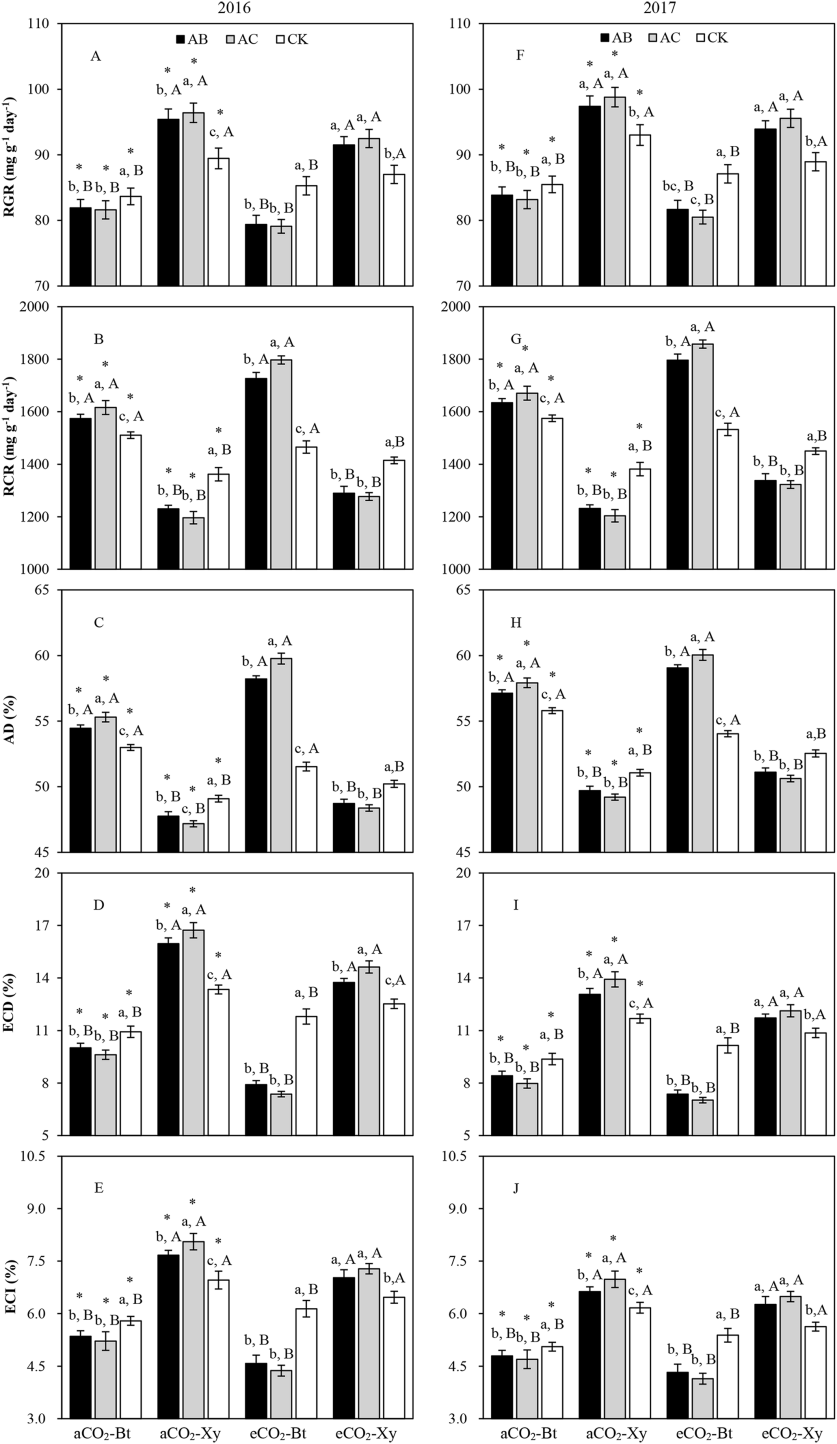
Impacts of the tri-interactions among CO_2_, transgenic treatment, and rhizobacteria infection on the food utilization of *M. separata* from the third to the sixth instar larvae in 2016 (A–E) and 2017 (F–J). Each value represents the average (+SE). Different lowercase and uppercase letters, and *indicated significant difference among three types of rhizobacteria infection for same type of maize under same CO_2_ level, between *Bt* maize and non-*Bt* maize for same type of rhizobacteria infection under same CO_2_ level, and between ambient and elevated CO_2_ for same type of maize and rhizobacteria infection by the Duncan test at *P* < 0.05 respectively.

There were opposite trends in the measured food utilization indexes of *M. separata* larvae fed on *Bt* maize (Bt) and non-*Bt* maize infected with *A. brasilense* (AB) and *A. chroococcum* (AC) compared with the CK in both years regardless of CO_2_ level (Fig. 4). In comparison with the CK, rhizobacteria infection with *A. brasilense* and *A. chroococcum* both significantly decreased RGR, ECD, and ECI of *M. separata* fed on *Bt* maize, and significantly increased RGR, ECD, and ECI of *M. separata* fed on non-*Bt* maize under the same CO_2_ level; and rhizobacteria infection with *A. brasilense* and *A. chroococcum* both significantly enhanced RCR and AD of *M. separata* fed on *Bt* maize, and significantly reduced RCR and AD of *M. separata* larvae fed on non-*Bt* maize under the same CO_2_ level. Moreover, compared with ambient CO_2_, elevated CO_2_ significantly increased RCR and AD, and significantly decreased RGR, ECD, and ECI of *M. separata* larvae fed on same type of maize cultivar infected with *A. brasilense* and *A. chroococcum* in both years (*P* < 0.05; Fig. 4). Furthermore, there were significant decreases in RGR, ECD, and ECI, and significant increases in RCR and AD of *M. separata* larvae fed on *Bt* maize in contrast to non-*Bt* maize infected with same type of rhizobacteria species within the same CO_2_ level in both years.

## Discussion

Insects are sensitive to environmental variations, and environmental stresses can cause changes on their growth, development, fecundity, food utilization and the occurrence and distribution of populations as a result of metabolic rate fluctuation (Bloom et al., 2010). In this study, elevated CO_2_ significantly prolonged larval and pupal duration and decreased pupation rate and pupal weight of *M. separata* compared to ambient CO_2_. Elevated CO_2_ negatively affected the larval survival, weight, duration, pupation, and adult emergence of cotton bollworm, *H. armigera* (Akbar et al., 2016), and reduced the egg laying by Cactus moth *Cactoblastis cactorum* (Stange, 1997) and *Achaea Janata* (Rao et al., 2013). In this study, elevated CO_2_ significantly increased the RCR (+10.44%) and the AD (+5.59%) (i.e., AD), and significantly reduced the RGR (−9.95%), ECD (−16.05%) and ECI (−17.95%) of *M. separata* larvae compared with ambient CO_2_. RGRs of Gypsy moth (*Lymantria dispar*) were reported to be reduced by 30% in larvae fed on *Quercus petraea* exposed to elevated CO_2_ (Hattenschwiler & Schafellner, 2004). RCR was significantly higher for *H. armigera* larva fed maize grown at 375 and 750 ppm CO_2_ in contrast to ambient CO_2_ condition, and elevated CO_2_ significantly decreased the ECI food, the ECD food, and the RGR of *H. armigera* larvae compared with ambient CO_2_ (Yin et al., 2010). According to the “Nutrition compensation hypothesis,” elevated CO_2_ can affect the development fitness of herbivores by changing the nutritional components, above and below-ground biomass, and photosynthetic rate of host plants indirectly (Ainsworth & Rogers, 2007; Jackson et al., 2009; Zavala, Nabity & Delucia, 2013), including increased C/N ratio and decreased nitrogen content etc. Declined growth rate, reproduction, and survival rate were found in the chewing mouthparts insects (e.g., *H. armigera*, *Spodoptera exigua*, *M. separata*), and the food consumption of which increased so that they could obtain necessary nutrition to survive (Bottomley, Rogers & Prior, 1993; Rogers et al., 2006). Yin et al. (2010) reported that elevated CO_2_ increased the food consumption and prolonged the development time of *H. armigera*, which due to the reduced nutritional quality of maize leaves, as a result of reduced nitrogen content and increased C/N ratio. Elevated CO_2_ significantly reduced the food conversion rate and enhanced the food ingestion of *H. armigera*, which attribute to reduced nitrogen content of the cotton, Simian-3 (Chen, Ge & Parajulee, 2005a; Chen et al., 2005b). Thus, Chen, Ge & Parajulee (2005a) and Chen et al. (2005b) inferred that elevated CO_2_ might be unfavorable to *H. armigera*. Our results in maize system appear to be similar to the study by Chen, Ge & Parajulee (2005a) and Chen et al. (2005b) in a cotton system.

Although the transgenic corn, *Zea mays* L., hybrids expressing the *Cry* insecticidal protein from *Bacillus thuringiensis* (Bt) were developed to control *H. zea, O. nubilalis, S. frugiperda*, and *M. separata* (Koziel et al., 1993; Armstrong et al., 1995; Jouanin et al., 1998; Lynch, Plaisted & Warnick, 1999), few studies focused on the defense responses of transgenic *cry1Ie* maize to corn armyworm under elevated CO_2_, especially on the growth, development and food utilization of the pest insects. Prutz & Dettner (2005) reported that the transgenic *Bacillus thuringiensis*-maize could result in decreased growth rate and increased mortality, which might attribute to the termination of larval metamorphosis. Most studies showed that adverse effects on life-table parameters of different herbivores were direct by the *Cry* protein (Lawo, Wäckers & Romeis, 2010), which might be due to the interaction of feeding inhibitors and growth inhibitors (e.g., secondary plant substances) (Smith & Fischer, 1983). Effects of elevated CO_2_ on the plant nutrition, metabolism and secondary defense metabolism might adverse for the growth, development and nutrition utilization of herbivores (Akbar et al., 2016). The insects possessed more nutrients to meet their growth needs and prolong the food digestion time in the midgut so that the RCR and AD increased (Reynolds, Nottingham & Stephens, 1985). In this study, we found that some negative effects of transgenic *cry1Ie* maize (Bt) and Xianyu 335 (Xy) grown in elevated CO_2_ on the food utilization indices (including RGR, ECD, and ECI) of *M. separata* larvae and some positive effects on the RCR and AD, which indicated that the resistance responses of *Bt* maize might persist under elevated CO_2_, and *M. separata* might ingest more food to get enough nutrition for surviving in limited developmental time under elevated CO_2_. Meanwhile the *Bt* maize and its parental line (Xianyu 335) prolonged their larval life-span and pupal duration, decreased growth rate and increased mortality that might result in lowering of pests’ occurrence. According to the “carbon nutrition balance hypothesis” (Gebauer, Strain & Reynolds, 1997), elevated CO_2_ would increase the fixed organic matter in plant while increase C-based secondary metabolites and decrease N-based secondary metabolites, thus affecting the insects resistance of plants. Robinson, Ryan & Newman (2012) indicated that elevated CO_2_ increased 19% phenols, 22% condensed tannins, and 27% flavonoids, while the terpenoids and NBSC decreased by 13% and 16% respectively. Coviella, Stipanovic & Trumble (2002) anticipated that the primary CO_2_ effect on *Bt* toxin production would be due to differences in N concentration within the plant. In a meta-analytical review of 33 studies that simultaneously increased CO_2_ conditions compared to ambient conditions, Zvereva & Kozlov (2006) showed that nitrogen concentration in plants was reduced under elevated CO_2_, and this decrease was stronger for woody compared to herbaceous plants. If conditions of increased carbon (e.g., elevated CO_2_) allow plants to allocate significantly more resources to condensed tannins and gossypol, then the enzyme composition in the insect herbivore is expected to also change. Similarly, if *Bt* toxin production changes due to elevated CO_2_, then the insect herbivore’s body enzymes should also be changed in this circumstance.

Most of the nitrogen, however, is found in the form of N_2_ which approximately amounts to 78% in the atmosphere. As plants cannot use this form of nitrogen directly, some microbes can change the N_2_ into ammonia. Most free living microbes in soil which can fix nitrogen and whose activities in enhancing the growth of plants are bacteria namely *Azotobacter* sp. and *Azospirillum* sp. These two bacteria are particularly important in maize production system due to their greater nitrogen fixing ability. Azospirillum acquires carbohydrate directly from sieve tube as a resource of carbon which promotes its growth (Olivera et al., 2004). Azospirillum can be used to promote the growth of sprouts under normal and arid conditions (Alejandra et al., 2009). Azospirillum also provides more flexibility to cell wall which enhances the growth (Pereyra et al., 2010) and increases products of wheat in waterless plot of land (Martin, 2009). Furthermore, azospirillum had the highest efficiency in nitrogen fixation at the root of sweet corn and it would reach the highest point of nitrogen fixation in the week 4 amounting to 0.20 mgNhr^−1^m^−2^ (Toopakuntho, 2010). Azospirillum can also create auxin, a substance promoting growth of maize, of 53.57 mg/ml (Phookkasem, 2011). Therefore, we used techniques of rhizobacteria (*A. brasilense* and *A. chroococcum*) inoculation of maize seeds to stimulate plant N uptake to increase in biomass N relative to C under elevated CO_2_, increase *Bt* toxin production for transgenic *cry1Ie* maize and create a substance promoting maize plant growth. In this study, we found that elevated CO_2_ significantly enhanced the rhizosphere soil densities both *A. brasilense* and *A. chroococcum* at the maize harvest, but there was no significant difference of the rhizosphere soil densities both *A. brasilense* and *A. chroococcum* between elevated and ambient CO_2_ at the maize seedling after 14 days. We hypothesize that the elevated CO_2_ increased the maize root bifurcation and soil nutrition (e.g., carbohydrates, amino acids and multi-trace elements) for rhizobacteria to provide the living space and nutrition with a long-time environmental effect. Other researchers have also shown positive effects of elevated CO_2_ on the bacterial community in the rhizosphere of maize (Chen et al., 2012). Moreover, significant adverse effects on the growth, development, reproduction, and food utilization of *M. separata* were observed when the host substrate maize was exposed to rhizobacteria treatments, which might be attributed to rhizobacteria stimulating plant N uptake to increase *Bt* toxin production for transgenic *cry1Ie* maize and promoting growth of its parental line (Xianyu 335) (Olivera et al., 2004; Stitt & Krapp, 1999).

There was no significant year-to-year variation in our field research data. Therefore, the overall results clearly indicate that increasing CO_2_ had negative effects on *M. separata*. Resistance performance of transgenic *cry1Ie* maize decreased under elevated CO_2_ as shown by decreased RGR, ECD, and ECI. The rhizobacteria treatments (*A. brasilense* and *A. chroococcum*) had positive effects on improving the effectiveness of *Bt* maize on target Lepidoptera pest management via decreased RGR, ECD, and ECI of *M. separata* that fed on transgenic *cry1Ie* maize and promoting growth of Xianyu 335 via increased RGR, ECD, and ECI of *M. separata*. Under future predicted climate changes (e.g., elevated CO_2_), it is particularly important to understand the field insect resistance traits of resistant crops to target pests. In an environment of accelerated greenhouse effect, *Bt* maize may have decreased resistance performance in the field with inhibiting effect on the development and food utilization of insects. Therefore, we used techniques of rhizobacteria (*A. brasilense* & *A. chroococcum*) inoculation of maize seeds to stimulate plant N uptake to increase in biomass N relative to C under elevated CO_2_, increase *Bt* toxin production for transgenic *cry1Ie* maize, and create a substance promoting maize growth.

## Conclusion

Overall, our results indicated that elevated CO_2_ and *Bt* maize were negative against development and food utilization of *M. separata.* Rhizobacteria infection significantly increased the larval life-span, pupal duration, RCR and AD of *M. separata*, and significantly decreased RGR, ECD and ECI of *M. separata* fed on *Bt* maize; there were opposite trends in development and food utilization of *M. separata* fed on non-*Bt* maize infected with rhizobacterias compared with the CK in 2016 and 2017 regardless of CO_2_ level. This study demonstrates that the use of rhizobacteria (e.g., *A. brasilense* and *A. chroococcum*) as pest control enhancer especially under elevated CO_2_ is significantly more beneficial in transgenic *Bt* maize system compared to that in non-transgenic system. Rhizobacteria (*A. brasilense* & *A. chroococcum*), as being one potential biological regulator to enhance nitrogen utilization efficiency of crops, could make the *Bt* maize facing lower field hazards from the target pest of *M. separate*, and finally improve the sustainability and resistance of *Bt* maize against target lepidoptera pests, especially under future CO_2_ raising.

## Supplemental Information

10.7717/peerj.5138/supp-1 Supplemental Information 1 Raw data.

## Additional Information and Declarations

### Data Availability

The following information was supplied regarding data availability: The raw data are provided in a Supplemental File.
